# Influencing factors in the nurses’ decision-making process in Ibero-American university hospitals

**DOI:** 10.1590/1518-8345.5648.3527

**Published:** 2022-05-25

**Authors:** Gilberto Tadeu Reis da Silva, Ises Adriana Reis dos Santos, Marimeire Morais da Conceição, Rebecca Maria Oliveira de Góis, Alexandre Silva Santos, Simone Coelho Amestoy, Renata Alessandra Evangelista, Maria Sagrario Gomez Cantarino, Alexandre de Assis Bueno, Paulo Joaquim Pina Queiros

**Affiliations:** 1 Universidade Federal da Bahia, Escola de Enfermagem, Salvador, BA, Brasil.; 2 Bolsista do Conselho Nacional de Desenvolvimento Científico e Tecnológico (CNPq), Brasil.; 3 Bolsista da Fundação de Amparo à Pesquisa da Bahia (FAPESB), Brasil.; 4 Universidade Federal de Pelotas, Escola de Enfermagem, Pelotas, RS, Brasil.; 5 Universidade Federal de Catalão, Departamento de Enfermagem, Catalão, GO, Brasil.; 6 Universidad de Castilla-La Mancha, Escuela de Enfermería y Fisioterapia, Toledo, Castilla-La Mancha, Espanha.; 7 Escola Superior de Enfermagem de Coimbra, Unidade de Investigação em Ciências da Saúde, Coimbra, Coimbra, Portugal.

**Keywords:** Health Services Administration, Nursing, Management Service Organizations, Decision Making, Leadership, Professional Autonomy, Administração dos Serviços de Saúde, Enfermagem, Organizações de Serviços Gerenciais, Tomada de Decisões, Liderança, Autonomia Profissional, Administración de los Servicios de Salud, Enfermería, Organizaciones de Gestión de Servicios, Toma de Decisiones, Liderazgo, Gestión en Salud, Autonomía Profesional

## Abstract

**Objective:**

to analyze the factors that influence nurses’ decision-making process in Ibero-American university hospitals.

**Method:**

a case study with a qualitative approach and of the multicenter type, carried out with 30 Ibero-American nurses. The data were collected through semi-structured interviews, analyzed by thematic categories and interpreted according to the theoretical framework of *Creating Effective Organizations*.

**Results:**

it was identified that the decision-making process permeates the development of nurses’ own competencies, suffering influences from health management training and previous individual experiences. The following categories emerged: *Technical-scientific preparation in decision-making; Hierarchization of the decision-making process*; and *Autonomous professional practice*.

**Conclusion:**

the absence/presence of a rigid hierarchy, as well as technical-scientific preparation and autonomy, are factors that limit or expand the range of possibilities in nurses’ decision-making, with consequences in care management. Thus, discussions about this theme should be encouraged, in order to promote nurses’ autonomy for decision-making and favor a reduction of bureaucracy in the processes that prevent/hinder advances in these services.

## Resumo

**Objetivo:**

analisar os fatores que influenciam o processo decisório de enfermeiros em hospitais universitários ibero-americanos.

**Método:**

estudo de caso, com abordagem qualitativa, do tipo multicêntrico, realizado com 30 enfermeiros ibero-americanos. Os dados foram coletados por meio de entrevistas semiestruturadas, analisados por categorias temáticas e interpretados de acordo com o referencial teórico da obra *Criando Organizações Eficazes*.

**Resultados:**

identificou-se que o processo decisório permeia o desenvolvimento de competências próprias dos enfermeiros, sofrendo influências da formação em gestão em saúde e de experiências individuais prévias. Emergiram as categorias: *Preparo técnico-científico na tomada de decisão; Hierarquização do processo decisório;* e *Prática profissional autônoma*.

**Conclusão:**

a ausência/presença de rígida hierarquização, bem como de preparo técnico-científico, e a autonomia são fatores que limitam ou ampliam a gama de possibilidades na tomada de decisão de enfermeiros, com reflexos na gestão do cuidado. Assim, discussões acerca dessa temática devem ser fomentadas, no intuito de promover a autonomia dos enfermeiros para a tomada de decisão e favorecer a desburocratização dos processos que impedem/dificultam os avanços nesses serviços.

## Resumen

**Objetivo:**

analizar los factores que influyen en el proceso de toma de decisiones de los enfermeros en hospitales universitarios iberoamericanos.

**Método:**

estudio de caso, con enfoque cualitativo, de tipo multicéntrico, realizado con 30 enfermeros iberoamericanos. Los datos fueron recolectados mediante entrevistas semiestructuradas, analizados por categorías temáticas e interpretados según el marco teórico de la obra *Diseño de Organizaciones Eficientes*.

**Resultados:**

se identificó que el proceso de toma de decisiones permea el desarrollo de las competencias de los enfermeros, se ve influenciado por la formación en gestión en salud y las experiencias individuales previas. Surgieron las siguientes categorías: *Preparación técnico-científica en la toma de decisiones*; *Jerarquización del proceso de toma de decisiones*; y *Ejercicio profesional autónomo*.

**Conclusión:**

la ausencia/presencia de una jerarquización rígida, así como la preparación técnico-científica y la autonomía son factores que limitan o amplían el abanico de posibilidades en la toma de decisiones de los enfermeros, que se reflejan en la gestión del cuidado. Por lo tanto, hay que incentivar las discusiones sobre este tema, con el fin de promover la autonomía de los enfermeros para la toma de decisiones y favorecer la reducción de la burocracia en los procesos que impiden/dificultan el avance de estos servicios.

**Highlights**

(1) Tools that influence nurses’ decision-making process are pointed out.

(2) Decision-making process restricted by the organizational hierarchical level they occupy.

(3) Need for investment in health management training.

(4) Importance of the scientific technical contribution associated with the nurses’ experiences.

(5) In opposition, a decision-making power limitation was evidenced.

## Introduction

In the course of work-related activities, it is common for the nurse to have doubts about which management tools can be used for better decision-making and to favor autonomy and leadership^1^ ^-^ ^5^. Therefore, it becomes indispensable to understand which elements make up the decision-making process, as it is an instrument of managerial competence inherent to the Nursing profession, as well as to know its implications for work and for the quality of the services. 

Nurses’ autonomy depends directly on the work environment, so that cultural and organizational aspects of the institution can influence it, favoring or impairing the decision-making process of these professionals. In both cases, there are direct impacts on the health services and on the labor relationships (individual or collective)^6^.

By decision-making, it is understood the choice between two or more alternatives that enable the achievement of a given objective. An international study^7^ describes decision-making in the Nursing practice environment as a dynamic conceptual process, capable of affecting the results in the services. Thus, in the course of the work activities, it is necessary that these professionals know how to identify and use resources that contribute to correct and self-reflexive decisions. 

The settled organizational model can show both the presence of *professional bureaucracy*, characterized by democracy at the operational level, and of *mechanized bureaucracy*, which consists of an autocratic style. The presence of both models in the same organization can represent a hierarchical system of the work process^8^, which also applies to the hospital environment. 

Institutions with *mechanized bureaucracy* properties are pointed out as limiting the communication between the different administration levels^9^, as they present a hierarchical, autocratic and vertical work mechanism, which provides the professional autonomy of a given category to the detriment of the work practice of another, in this case, of the nurse.

Moreover, in these institutions, nurses are not granted top management positions, due to lack of professional recognition, especially of their autonomy in the social, economic, political and legal instances^10^. However, considering that the main focus of these professionals’ practice lies in the implementation of activities, to achieve maximum effectiveness and productivity, which also need to be valued, it becomes indispensable to make hierarchical relationships more flexible, in order to allow for the participation of these professionals in the actions and decisions, as well as the opening of a dialog between the various actors involved. 

On the other hand, an organizational model with *professional bureaucracy* properties, implemented in health services, contributes to the development of managerial competences by nurses, such as leadership, communication and decision-making^9^, given the presence of less rigid protocols, which favor better working conditions. Thus, the focus of such bureaucracy is not related to the power struggle to obtain and predominate in a given professional category, but to the performance of actions thought, coordinated, integrated and shared with the group. 

In this managerial perspective, an organizational culture is noticed that prioritizes professional improvement and quality of the care provided to the target population^11^ ^-^ ^12^, due to recognition of the work performed, of the engagement and of the development of autonomy for decision-making by nurses. In addition to that, this culture favors changes in behaviors and performance scenarios; in this case, university hospitals. It is worth mentioning that these hospitals are recognized as spaces to support teaching, research and extension, linked to higher education institutions responsible for training health professionals. 

In summary, given the importance of identifying how the decision-making process takes place in the hospital environment and its interface with the work of nurses, the question is: which factors influence nurses’ decision-making process in Ibero-American university hospitals? 

To answer this question, the objective was to analyze the factors that influence nurses’ decision-making process in Ibero-American university hospitals. To support the analysis, the theoretical framework of *Creating Effective Organizations* ^9^ was adopted, which addresses the dynamics of the organizations. Influence was observed as a contingency factor in the *authority flows*, in the control of human behavior and in the *information flows* related to the decision-making processes; and decision-making, as an organizational characteristic in health institutions.

As for the relevance of the research, the intention was to encourage reflection on the Nursing management practice in the hospital context, in order to contribute to the development of studies in this area. It was also intended to boost the state-of-the-art on the theme and to subsidize the implementation or reconfiguration of the management models, in order to stimulate the role of nurses in such spaces. 

In addition, it is intended to add knowledge in Nursing, through the approximation of the results identified, interpreting them in the light of the theoretical perspectives of the administration area, such as the case of the *mechanized and professional bureaucracies* conceptions, which can contribute to the understanding of the dynamics of organizations and of management in the health area, with a view to facilitating nurses’ managerial behaviors.

## Method

### Study design

A case study with a qualitative, descriptive and exploratory approach and of a multicenter character, having the *think-see-do* triad as theoretical framework^9^, where decision-making is related to rational, intuitive and improvised actions. In addition, in health, application of these concepts allows for a fresh perspective at the organizational configuration, in the presence of elements capable of interfering with the success of reforms in health systems.

The *Consolidated Criteria for Reporting Qualitative Research* (COREQ) instrument was used to structure the data^13^.

### Scenario and participants

This is a research study conducted in university hospitals from three Ibero-American countries: one in the Northeast region of Bahia, Brazil; another one in Coimbra, Portugal; and the third one in Toledo, Spain. 

In Brazil, university hospitals have a linear/hierarchical organizational structure, which follows a clear command chain. These hospitals are federal government entities, maintained by the Ministry of Education (MEC) and the Unified Health System (*Sistema Único de Saúde*, SUS), which, since 2011, are under the administration of the Brazilian Hospital Services Company (*Empresa Brasileira de Serviços Hospitalares*, EBSERH)^14^. 

In Spain and Portugal, university hospitals also follow a structural alignment, as is the case in Brazil. They are public hospitals, managed by the National Health Services. However, there is a contractual relationship for the provision and consideration of services, supervised by the regional public sector, with internal administration and autonomous bodies in each of these countries.

We emphasize that, in line with the studies by these authors^15^ ^-^ ^16^, it is necessary to clarify that, since the 1990s, the Iberian countries have implemented public policies and investments in excellence programs for the management of hospital services linked to universities. This investment is intended for the training of professionals to qualify in health management. The public policies referred to are focused on health protection as a citizens’ right through public and universal health systems.

Thus, the legislation in force both in Spain and in Portugal has principles and rules applicable to the health units, which calls on university hospitals to assume their nature as public business entities, made explicit in their strategic plan. In this case, they must strive to achieve the pre-established goals and to measure the results.

### Participants

A total of 30 nurses participated in the research: eight from Brazil, nine from Spain and 13 from Portugal. All of them developed managerial activities at the micro-, medium- or macro-organizational level in care and/or administrative areas. 

### Selection criteria

The study included nurses who had been working in the services for at least one year. Choice of these participants resulted from the significant experience they had with decision-making in the service, which enabled them to give a more accurate opinion in this regard. Nurses on leave or vacation or who did not attend three interview attempts were excluded from the study.

### Instruments used for collecting the information

The technique chosen for data collection was individual interviews through the application of a semi-structured questionnaire, elaborated and validated by specialists in Portugal and Brazil. Such questionnaire contained 12 closed questions, with diverse information about the sociodemographic profile (age, gender, marital status and schooling), and three open questions, each with five sub-questions related to the organizational management model, five related to the Nursing management model, and two related to management practices and instruments.

As for the tool used to select the participants, we opted for the *snowball sampling* technique^17^, which uses reference chains from a group called seed. Thus, the approach of these participants took place through a formal invitation to participate in the research, both in person and through telephone contacts and/or sending short messages and brief texts via cell phone or email, known as WhatsApp. This technique was chosen due to the difficulty accessing to the researched population, due to the work routine in the function performed and/or the positions held by these professionals. It is worth mentioning the adoption of the data saturation criterion for completion of the collection process, that is, the interruption at the time when there were no new contacts and the information was only repeated.

All interviews were recorded with the aid of a smartphone mobile device. 

### Period and data collection

The research was conducted from September 2019 to February 2020, and the data were collected through individual interviews, with a mean duration of 90 minutes each. They took place in a place, date and time previously established by the professionals and were conducted by researchers (responsible professor and PhD students) previously prepared. 

All participants were duly instructed about the research and expressed their consent to participate by signing the Free and Informed Consent Form (FICF) in two copies (participant and researcher). They were also informed about the possibility of withdrawing from participation at any time, without any consequences. It is worth mentioning that the subjects took part in all the research stages.

The recordings of the interviews were authorized by signing the Declaration of Assignment of Rights on Oral Statements. After full transcription of the interviews, they were returned to the participants for content validation and/or correction.

In order to preserve anonymity, the participants were identified by means of flower names: Sunflower; Clove; and Violet for the Brazilian, Spanish and Portuguese participants, respectively. Numbers corresponding to the order in which the interviews were conducted were added to the codes: Sunflower 1 to 8; Clove 1 to 9; and Violet 1 to 13. Choice of these codenames was suggested by one of the nurses who participated in the study. 

### Data treatment and analysis

The data were treated and analyzed through content analysis^18^. Use of this technique enables replication and validation of the inferences about a given phenomenon, with application of specialized and scientific procedures. It was initiated with the *organization of the materials*, the sorting stage, attentive and deep reading of the texts and references used to mark or complement the diverse information of the study, along with the notes described by the researchers and the transcripts of the interviews. Subsequently, *thematic categorization was carried out with the search for units of meaning*, in which, from the careful re-reading of the material, the data were clipped and organized in a table prepared in Word. Thus, according to the relevance of the subject matters presented, they were classified/typified into drawers. In the end, three thematic categories emerged: *Technical-scientific preparation in decision-making; Hierarchization of the decision-making process*; and *Autonomous professional practice*. Subsequently, the *deepening and interpretation of the findings* stage was initiated in the light of the theoretical framework^9^ adopted, more specifically about the *simple structure of the organization, with the mechanical and professional bureaucracies*. 

The data were managed with the aid of the Nvivo^®^11 and WebQda^®^ software programs.

### Ethical aspects

This research was approved by the Research Ethics Committee (*Comitê de Ética em Pesquisa*, CEP) of the Nursing School of the Federal University of Bahia, under opinion No. 3,374,244. All the recommendations set forth in Resolutions No. 466/2012 and No. 510/2016 of the National Health Council were followed.

## Results

**Figure 1:**
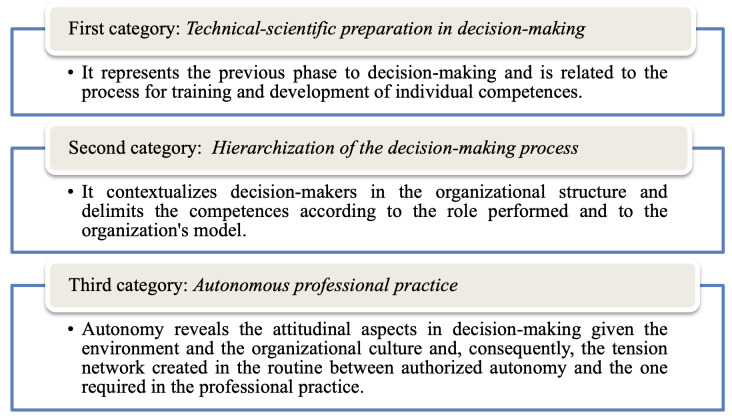
Description of the moments

Data analysis evidenced some factors that influence nurses’ decision-making process in Ibero-American university hospitals. These aspects were unveiled through the elaboration of three thematic categories, which, when carefully observed, represent different moments of the decision-making process (Figure 1).

*1) Technical-scientific preparation in decision-making*

The results present an interface with the three fundamental decision models identified by the theoretical framework, namely: rational, intuitive, and improvised decisions. The rational model can be characterized by “thinking first”; the intuitive indicates “seeing first”; and the “improvisational” is justified by “doing first”. Based on this approach, it is possible to synthesize the model for decision-making in the “think-see-do” triad.

Thus, scientific knowledge, management training and professional experience emerged as essential tools for good practices in decision-making by nurses, as evidenced in the excerpts from the following speeches: *I have no doubt that the University Hospital management has committed nurses, competent, with training in management, qualities, characteristics, behavioral skills [...] and leadership.... Because she can hire professional technicians, appoint someone, nominate, it becomes easier [...] and if she doesn’t have these requirements, she won’t be able to manage, but if she has them, I believe that it’s the ideal space for nurses and that she can improve many university hospitals in the country*(Sunflower 1)*. For me, it’s always been like this, decisions always have to be based on scientific knowledge, a lot of experience is also necessary, but decisions have to be based on scientific knowledge*(Violet 7).

*2) Hierarchization of the decision-making process*

A number of theoretical assumptions indicate that the hierarchy of the decision-making process can happen implicitly, as an integral part and/or result of the organizational structure, depending on how coordination and division of work are conducted by the managers. 

On the other hand, the practice of the decision-making process by nurses expresses a limitation in the capability of these professionals to decide, as it is under the aegis of a specific management model, which can exert an impact on the services and on staff shortages. Some excerpts evidence this facet: *Regarding power, we also don’t have enough, that is, we’re just a transmission belt, so there’s no movement towards autonomy. From the moment that the boards of the intermediate management units negotiate, subscribe, sign and agree with the program contract, for example, the 2020 contract, and in this program contract all the necessary means and the results are detailed, obviously what we intend to achieve, the middle management teams should have the power to make contracts, to authorize additional work, to hire another nurse to replace the one who retires*(Violet 2).

*In line with the above report and with the analysis framework, we identified the existence of a vertical command chain, associated with the organizational model, in which the participation of nurses in decision-making follows the labor relationships and/or the work regime put into practice. This can be seen in the following excerpts from the interviews: Decision-making processes are centralized, hierarchical [...]. We had management over the employee hired by the foundation to support research in university hospitals, this experience has been better. Because we followed a more simplified selection process and chose the individual according to the need for the service*(Sunflower 4)*. The management of health services, in a general way, as you look at the nurses’ decision process, as I see it at the hospital level is very vertical, that is, from top to bottom, the Nursing head and the adjuncts, the adjuncts are like the supervisors, they follow a vertical line that is very, very hierarchically vertical, an assertive style is what the head says and the adjuncts do downwards*(Clove 4).

*3) Autonomous professional practice*

According to the theoretical framework, the decision-making process represents a set of actions and dynamic factors that begins with the identification of a stimulus, which requires action and ends with a decision, that is, it requires the implementation of a decision-making process, either formal or informal, structured or unstructured. The first stage of this process is the recognition and analysis of a problem or opportunity, which will guide the decisions. Subsequently, the objectives that will establish the alternatives are defined.

In this context, the professional autonomy of the groups depends on and is determined by the ability of central managers to listen and accept the suggestions made by these professionals, who are in direct contact with the patients, as their amplitude is conditioned to the amount of resources available for decision-making. This aspect can be observed in the some participants’ statements: *[...] the Nursing management is very good [...]. Despite this medical care model, we still have a lot of autonomy here. We have a lot of leadership, a lot of voice. The staff listens a lot to what we, who are with the patient daily, have to say*(Sunflower 9)*. [...] I reassert having autonomy, because you can decide my work process, you can decide that it’s better for the population, for the citizens and for everyone. I have autonomy in my office and all the nurses too, [...]* (Clove 7)*. Nurses have an autonomous position; therefore, they’re care-related decisions that they obviously describe and that listen to the patients. Meanwhile, as head nurse, you have to promote this critical sense (in the sense of a correct analysis of the user’s needs) and, thus, identification of these needs and then the autonomous prescription of what Nursing care is. Obviously, nurses have interdependent roles and therefore assume responsibilities at the same level as the autonomous roles, for being in charge of the care provided to the patient as a whole and, therefore, having an independent, autonomous, rigorous form of responsibility of involvement competence all the same*(Violet 4).

## Discussion

Development of autonomy was evidenced in this study as a process influenced by individual aspects and by the characteristics of the organization. In this case, predominance of a hierarchical management model was identified, which directly interferes in decision-making quality. 

Based on the *think-see-do* triad^9^, which includes rational, intuitive and improvised actions for decision-making, it is noticed that, given the complexity of the services offered, skills development by nurses is linked to an eventual investment by the institution in health management training^19^; applied in the services as a contribution to work performance, with the joint use of technical-scientific knowledge and experiences underwent by these professionals. 

The possibility of achieving better and more significant results in the services managed by nurses is also reinforced by the presence of professionals with high *know-how* abilities to adopt critical thinking in favor of decision-making^19^ ^-^ ^23^. 

Nevertheless, in *professional bureaucracy* ^9^ there is a decentralized and specialized structure, supported by standardized coordination, mediated by *training* (knowledge and skills acquired in training) and *indoctrination* (use of formal knowledge, integrated into the practice experienced in the service). This makes it possible for all the actors involved to operate the services independently from other professionals and closer to the clients. 

In this sense, the hierarchization model of the decision-making process adopted by the institution can represent a limitation or a possibility for the managers^22^. If nurses’ decision-making includes the elaboration of service rosters and staff sizing, but excludes the effective hiring of the Nursing team, the limitation of the autonomy exercised by these professionals in the decisive instance for solving the problem is made evident, with consequences in work overload, quality management and resolution, among others. 

Thus, the presence of autonomy limiting factors is related to the institutional management model, and this will be decisive for the managerial actions in Nursing to be characterized as independent, interdependent or dependent. 

Non-resolution for these demands will result in work overload, in the face of a context of personnel shortage, which, in turn, will compromise professional satisfaction and, therefore, the care quality offered^22^ ^-^ ^28^. A research study carried out in Spain^27^ revealed the impact of instrumentalizing health in the nurses’ practice and in the presence of a management model with rigid and inflexible standards. Furthermore, a survey on the relationship between autonomy and moral distress in nurses working in emergency services from Iran^28^ indicates that the reduction of professional independence impairs decision-making and the ability to implement appropriate interventions.

Such aspects are in line with the concepts of mechanized bureaucracy^9^, whose organizational structure follows normative standards for the work process and presents a formal chain for decision-making. In this sense, it can be asserted, as also verified in the current study, that this bureaucracy is still strongly present in hospital institutions. Thus, reflecting on their ties can be the first step to transpose rigid behaviors in these work environments. 

In Brazil, these hospitals are maintained by the Federal Government, although labor relationships usually include two or more employment relationships (CLT contracted, statutory and outsourced). Thus, it is assumed that the decision-making process of the manager nurse and/or of the services meets the specific regime of the hired employee. 

It is worth mentioning that the Brazilian Constitution does not recommend the use of outsourcing in core activities, due to the harms caused by the precarious relationship of health professionals, who, in general, earn low salaries and work long and exhausting hours. This is a situation that significantly compromises the work process^14^ ^,^ ^29^ ^-^ ^30^. 

An international study^31^ identified lack of recognition, salary inequality and distinctions between permanent and temporary nurses who perform the same functions. In addition to that, it was found that, eventually, these professionals had some/no participation in the decision-making process. Such conditions impair the routine of the services that present this multiplicity of links and act as a demotivating element for the professional performance and compliance with the standardized routines, as there are disparities in the treatment modality offered to the professionals inserted in this context. 

However, in opposition to the structural model advocated by *mechanized bureaucracy* ^9^, some organizations present *professional bureaucracy* characteristics^9^ and, in them, the professionals enjoy certain autonomy and freedom for decision-making. In these environments, not only the quality of the services provided is favored, but also patient safety. 

Therefore, the health organization’s responsibility to promote and provide means for nurses to act autonomously, through the establishment of roles, responsibilities and behaviors, is clearly emphasized^31^ ^-^ ^37^. In line with the professional bureaucracy^9^, it becomes a priority to choose the development of leaders in Nursing as the institution’s strategic objective. At the same time, union between nurses in the search for autonomy should be encouraged, as well as the commitment to assume the responsibilities resulting from such autonomy. 

In summary, the results herein presented evidence similarities between the realities experienced by Ibero-American nurses. Therefore, transforming this reality is a common concern and needs to be discussed together with these professionals. 

It is suggested to integrate these professionals through the creation of dialogical spaces (virtual/face-to-face), in order to socialize experiences about the nurses’ role/performance in these three countries. In addition to that, it is recommended to give these professionals a voice with the top management, in order to unveil the specificities inherent to each university hospital and their respective management models. 

As contributions, the results of this research evidence the need to better understand the inclusion of Nursing in the health services. In this sense, although nurses’ professional performance has been objectively and notoriously defined throughout history, through the development of skills and abilities, it is noticed that preservation of their autonomy for the full professional practice at the time of their institutional insertion requires further discussions in order to recognize, value and preserve the professional identity. 

A limitation of this study lies in the fact that it was carried out only with university hospitals, as its results cannot be generalized or attributed to the category as a whole. 

## Conclusion

In this study, tools that influence the nurses’ decision-making process were identified. They are as follows: *Technical-scientific preparation*, *hierarchization* and *autonomy*. It is noteworthy that the theoretical precepts about organizational structures were the basis for the analysis of this research, correlated with the thematic categories elaborated. 

It was found that, many times, the decision-making process in the nurses’ professional practice is constrained by the level occupied by these professionals in the organizational hierarchy, something worthy reinforcing as quite characteristic of the *professional bureaucracy* model. However, it is noticed that, when permeated by the development of managerial skills by nurses, this environment is capable of promoting an advance in autonomy linked to the improvement of training.

With regard to the training of future Nursing professionals, due to their configuration (teaching, research and extension), university hospitals need to employ resources that provide support for the construction of autonomy and critical thinking, so that they can improve decision-making. In addition, it is recommended to invest in permanent and continuing education for the professionals inserted in this context.

The joint acquisition of technical-scientific knowledge and of practical experiences is highlighted as resources that contribute to the development of critical positioning during the decision-making process. Likewise, the importance is highlighted of the hospital manager’s participation as a facilitator of actions in the implementation of strategies that provide greater group engagement with the services and, consequently, in offering greater autonomy to nurses. 

Another point observed concerns the autonomous professional practice focused on direct patient care. On the other hand, there was a limitation of the decision-making power with regard to meeting the needs of the services, especially the management of the service schedule. 

This research analyzed factors influencing the nurses’ decision-making process during their professional practice in Ibero-American university hospitals and, therefore, was limited to studying the phenomenon through specific perspectives, in certain educational institutions. However, although the current analysis cannot be generalized to other contexts, it contributes to the reflection on the theme and indicates the need to favor elements such as the autonomy of professional nurses, in order to facilitate decision-making processes and reduce bureaucracy in the services. They are important measures that favor an agile and safe assistance dynamic in the hospital environment. 

For the purpose of comparing the results obtained, it is suggested that this study be replicated in other active scenarios of the basic network (outpatient clinics, specialized clinics), as well as that futures surveys include the other social actors that work in care management. It is also considered valid to apply methods to carry out cross-sectional and longitudinal studies that expand the range of observations and favor the compilation of answers to verify the phenomenon related to the decision-making process from a quantitative perspective.
